# Postoperatives Management

**DOI:** 10.1007/s00508-023-02272-7

**Published:** 2023-10-11

**Authors:** Verena Parzer, Michael Resl, Lars Stechemesser, Maria Wakolbinger, Bianca Itariu, Johanna Maria Brix

**Affiliations:** 1 1. Medizinische Abteilung mit Diabetologie, Endokrinologie und Nephrologie, Klinik Landstraße, Wien, Österreich; 2 ICMR – Institute for Cardiovascular and Metabolic Research, Johannes Kepler Universität Linz, Linz, Österreich; 3 Universitätsklinik für Innere Medizin I mit Gastroenterologie, Hepatologie, Nephrologie, Stoffwechsel und Diabetologie, Uniklinikum der Paracelsus Medizinischen Privatuniversität, Salzburg, Österreich; 4 Abteilung für Sozial- und Präventivmedizin, Zentrum für Public Health, Medizinische Universität Wien, Wien, Österreich; 5 Karl Landsteiner Institut für Adipositas und Stoffwechselerkrankungen, Klinik Landstraße, Wien, Österreich; 6 Abteilung für Innere Medizin mit Diabetologie, Gastroenterologie und Hepatologie, Rheumatologie und Intensivmedizin, Konventhospital der Barmherzigen Brüder Linz, Linz, Österreich; 7 Klinische Abteilung für Endokrinologie und Stoffwechsel, Universitätsklinik für Innere Medizin III, Medizinische Universität Wien, Wien, Österreich

**Keywords:** Adipositas, Mikronährstoffe, Dumping-Syndrom, Typ-2-Diabetes, Komplikationen, Obesity, Micronutrients, Dumping syndrome, Type 2 diabetes, Complications

## Abstract

Die bariatrische Chirurgie führt zu einem signifikanten Gewichtsverlust, einer Reduktion oder gar Remission einer Vielzahl an Adipositas-assoziierten Begleiterkrankungen, einer Reduktion der Mortalität und einer Verbesserung der Lebensqualität vieler PatientInnen. Dennoch ist Adipositas eine chronische Erkrankung, die auch nach bariatrischer Operation eine Weiterbetreuung erforderlich macht. Zudem können kurz- oder langfristig spezifische Probleme auftreten, wie beispielsweise Mangelzustände verschiedener Mikronährstoffe und damit assoziierte Komplikationen. Bauchschmerzen sind ein immer ernst zu nehmendes Symptom nach bariatrischen Operationen. Ein weiteres Augenmerk sollte auf der Diagnose und Behandlung des Dumping-Syndroms liegen. Personen mit Typ-2-Diabetes sollen auch bei substanziell verbesserter bzw. normalisierter Glykämie regelmäßig auf wiederkehrende Hyperglykämie und spezifische Folgeerkrankungen gescreent werden. Neben spezialisierten Zentren mit multidisziplinären Teams wird der Primärversorgung und hier insbesondere ÄrztInnen für Allgemeinmedizin eine zunehmend wichtige Rolle in der Nachsorge nach bariatrischen Operationen zukommen.

## Abstract (en)

Bariatric surgery results in significant weight loss, reduction or even remission of obesity-associated comorbidities, reduced mortality, and improved quality of life in many patients; however, obesity is a chronic disease, thus follow-up care is required after bariatric surgery. Furthermore, specific issues, such as micronutrient deficiencies and subsequent complications, can arise both in the short-term and the long-term. Abdominal pain after bariatric surgery must always be regarded as a serious symptom. A further focus should be on the diagnosis and treatment of dumping syndrome. Patients with type 2 diabetes should be regularly screened for recurrent hyperglycemia as well as specific sequelae, even though blood glucose levels may be substantially improved or normalized. In addition to centers with multidisciplinary teams, primary care and, in particular, general practitioners will play an increasingly more important role in the follow-up care after bariatric surgery.

Nach bariatrischen Eingriffen wird eine multidisziplinäre Langzeitnachsorge empfohlen, und die Bereitstellung eines angemessenen Nachsorgeprogramms durch ein multidisziplinäres Team ist für bariatrische Zentren essenziell. Dieses multidisziplinäre Team sollte sich aus folgenden Professionen zusammensetzen: FachärztIn für Innere Medizin mit Adipositas-Schwerpunkt bzw. AllgemeinmedizinerIn mit Zusatzausbildung im Bereich Adipositas, ChirurgIn, DiätologIn oder ErnährungswissenschafterIn, SpezialistIn für körperliche Aktivität wie Physio- oder SporttherapeutIn, PsychiaterIn oder PsychologIn, KrankenpflegerIn und AllgemeinmedizinerIn der/des PatientIn. Darüber hinaus können Adipositas-SpezialistInnen, DiätologInnen sowie Pflegepersonen, die nicht speziell in der bariatrischen Medizin ausgebildet sind, in der beruflichen Tätigkeit mit bisher unbekannten Problemen aufgrund eines bariatrischen Eingriffes bei ihren PatientInnen konfrontiert werden.

Eine Überweisung an ein bariatrisches Zentrum ist oft notwendig und sollte möglich sein, jedoch besteht auch ein wachsender Bedarf an der Verbreitung von Kenntnissen im Umgang mit bariatrischen PatientInnen. Vor allem bei komplexeren klinischen Situationen sollte eine Überweisung an ein bariatrisches multidisziplinäres Zentrum – vorzugsweise jenes, das den ursprünglichen Eingriff vorgenommen hat – in Betracht gezogen werden. Bariatrische PatientInnen können nach der Operation mit neuen, spezifischen und vielschichtigen klinischen Problemen konfrontiert sein: Die Essgewohnheiten müssen an die neue gastrointestinale Physiologie angepasst werden, und je nach Art des bariatrischen Eingriffs können Ernährungsdefizite auftreten. Die Behandlung von Krankheiten, die mit Adipositas in Zusammenhang stehen, muss an den Gewichtsverlust angepasst werden, wobei mögliche Änderungen der Pharmakokinetik von Medikamenten zu berücksichtigen sind. Besondere Probleme können bei Frauen während der Schwangerschaft auftreten. Des Weiteren können PatientInnen psychologische Schwierigkeiten haben, sich an die tiefgreifenden Veränderungen des Essverhaltens und des Körperbildes anzupassen. Schließlich kann es zu einer erneuten Gewichtszunahme kommen, die verhindert und behandelt werden sollte [1].

In Anbetracht der steigenden Zahl von PatientInnen mit bariatrischen Eingriffen sollte die Nachsorge jedoch im Laufe der Zeit zumindest teilweise in die Primärversorgung verlagert werden [1]. Bei der Behandlung und Untersuchung in der Primärversorgung sind ÄrztInnen für Allgemeinmedizin die erste Anlaufstelle für Erwachsene mit Adipositas. Die kritischen Faktoren, die für AllgemeinmedizinerInnen in diesen Bereichen relevant sind, sind in Tab. 1 zusammengefasst und über die postoperative Nachsorge hinaus anwendbar [2].

**Table 1:** 

Verwendete Ansätze/Maßnahmen	Begründungen/Erläuterungen
Verbesserung von Kommunikation und Motivation	Motivation ist für die Therapietreue von entscheidender Bedeutung; die Bereitschaft zur Veränderung wird langfristig mithilfe des motivierenden Interviews (Motivational Interviewing [MI]) bewertet
Stigmatisierung in der Gesundheitsversorgung vermeiden	Stigmatisierung ist im Gesundheitswesen sehr häufig. Die Folgen sind eine Zunahme von Essstörungen, die den Grad der Adipositas verschlimmern, sowie eine Zunahme von Depressionen, Suizidgedanken oder im schlimmsten Fall sogar von Suizid; die Stigmatisierung kann durch MI verringert werden
Messung des Taillenumfangs	Er ist ein guter Indikator für viszerales Fett und ein nützlicher Prädiktor für kardiometabolische Erkrankungen; er kann in regelmäßigen Abständen gemessen werden, um die Abnahme des viszeralen Fettes zu überwachen
Behandlung von Komorbiditäten	Komorbiditäten sollten vorrangig behandelt werden, v. a. die kardiometabolischen Erkrankungen, um die Mortalität zu senken
Einsatz eines multidisziplinären Teams	Ein multidisziplinäres Team (FachärztIn für Innere Medizin mit Adipositas-Schwerpunkt bzw. AllgemeinmedizinerIn mit Zusatzausbildung im Bereich Adipositas, ChirurgIn, DiätologIn oder ErnährungswissenschafterIn, SpezialistIn für körperliche Aktivität wie Physio- oder SporttherapeutIn, PsychiaterIn oder PsychologIn, KrankenpflegerIn und AllgemeinmedizinerIn der/des PatientIn) ist effizienter; dieses Team arbeitet in einem Netzwerksystem
Änderung des Lebensstils in Betracht ziehen	Verhaltensänderungen tragen dazu bei, das Körperbild, das Selbstwertgefühl, die Selbstbestätigung und die Lebensqualität zu verbessern
Mehr körperliche Aktivität	Regelmäßige körperliche Aktivität verringert das Risiko eines erneuten Gewichtsanstiegs nach einer Gewichtsabnahme
Vermeiden von Gewichtsschwankungen	Nach der Gewichtsabnahme wird besonders darauf geachtet, eine erneute Gewichtszunahme und Gewichtsschwankungen zu vermeiden. Die PatientInnen können sich etwa alle 2 Wochen wiegen; wenn die/der PatientIn schnell 3–4 kg zunimmt, sollte sie/er nicht zu lange warten, bevor sie/er die/den AllgemeinmedizinerIn aufsucht, um sich untersuchen zu lassen

## Kommunikation ohne Stigmatisierung

Kommunikation ist ein grundlegender Aspekt jedes Behandlungsverhältnisses, so auch der Adipositas-Behandlung. Die Art und Weise, wie mit den PatientInnen kommuniziert wird, ist entscheidend für den Aufbau eines guten therapeutischen Bündnisses. Die verschiedenen Ansätze zur Behandlung der Adipositas einschließlich der medikamentösen Gewichtsreduktion und der bariatrischen Chirurgie [3] beruhen auf einer guten Kommunikation mit den PatientInnen, die an Adipositas leiden, auf Motivierung zur Veränderung und therapeutischer Aufklärung der PatientInnen sowie auf objektiver klinischer Bewertung, Verhaltenstherapie und Vermeidung von Stigmatisierung [2].

## Versorgung mit Mikronährstoffen

Bei Menschen mit Adipositas können bereits präoperativ alterierte Blutwerte verschiedener Mikronährstoffe auffallen. Nach bariatrischen Operationen treten oftmals relevante Mängel von Mikronährstoffen auf [4, 5]. Eine strukturierte Nachsorge beinhaltet daher eine regelmäßige Labordiagnostik und Supplementierung sowie ggf. den Ausgleich eventueller Nährstoffmängel. Das Risiko für die Entstehung von Nährstoffmängeln ist bei rein restriktiven Operationsverfahren geringer als bei malabsorptiv wirkenden Verfahren und wird darüber hinaus von Diät und Essverhalten beeinflusst [6–8].

Das empfohlene Mikronährstoffmanagement nach bariatrischer Operation wird inklusive Symptomatik bei Mangel, empfohlener täglicher Zufuhr sowie Dosis einer Supplementierung und Aufsättigung in Tab. 2 zusammengefasst.

**Table 2:** 

Nährstoff	Symptome	Empfohlene Tagesdosis (RDA)	Supplementation	Aufsättigung
Vitamin A	Frühe Anzeichen: Nachtblindheit, Bitot-Flecken, Hyperkeratose, Geschmacksverlust	Männer: 900 µg (3000 IE)	RYGB, SG: 5000–10.000 IE/Tag	Keine Korneaveränderungen: 10.000–25.000 IE/Tag p.o. bis zur klinischen Verbesserung (1–2 Wochen)
	Anzeichen eines fortgeschrittenen Mangels: Korneaschäden (Xerophthalmie, Keratomalazie), Erblindung	Frauen: 700 µg (2300 IE)		Korneaveränderungen: 50.000–100.000 IE/Tag i.m. für 3 Tage, dann 50.000 IE/Tag i.m. für 2 Wochen
Vitamin D	Hypokalzämie, Kribbeln, Krämpfe, Tetanie, metabolische Knochenerkrankungen (Rachitis, Osteomalazie), Muskelschmerzen	Allgemein: 600 IE	3000 IE D3/Tag, um einen 25(OH)D-Spiegel > 30 ng/ml zu halten	3000–6000 IE D3/Tag (bevorzugt) oder 50.000 IE D2, 1 bis 3 Dosen/Woche
		Schwangerschaft, Stillzeit oder ≥ 71. Lebensjahr: 800 IE		
Vitamin E	Sensorische und motorische Neuropathie, Ataxie, retinale Degeneration, hämolytische Anämie	Allgemein: 15 mg (22,4 IE)	Erwachsene und Jugendliche ab 14 Jahren: 15 mg (22,4 IE)/Tag	90–300 mg (100–400 IE)/Tag
		Stillzeit: 19 mg (28,4 IE)	Stillzeit: 19 mg (28,4 IE)/Tag	
Vitamin K	Gerinnungsstörung, Hämorrhagie	90–120 µg	RYGB, SG: 90–120 µg/Tag	Akute Malabsorption: 10 mg Vitamin K parenteral
				Chronische Malabsorption: 1–2 mg/Tag p.o. oder 1–2 mg/Woche parenteral
Vitamin B_1_ (Thiamin)	Periphere Neuropathie: Taubheit, Kribbeln in den Extremitäten	1,5 mg	> 12 mg/Tag, bevorzugt 50–100 mg/Tag als B‑Komplex-Supplement	p.o.: 100 mg 2‑ bis 3‑mal/Tag bis zum Abklingen der Symptome
	Gangataxie, Erbrechen, Verwirrung		Bei i.v.-Flüssigkeitszufuhr 100 mg Thiamin zugeben (bei Verdacht auf Wernicke-Enzephalopathie keine Glukose!)	i.v.: 200 mg 3‑mal/Tag oder 500 mg 1‑ bis 2‑mal/Tag für 3 bis 5 Tage, dann 250 mg/Tag für 3 bis 5 Tage, gefolgt von Erhaltungstherapie mit 100 mg/Tag p.o. (unbegrenzt)
	Wernicke-Korsakoff-Syndrom: Enzephalopathie, Ataxie, okulomotorische Dysfunktion, Gedächtnisstörungen, Konfabulation, Lernstörungen			i.m.: 250 mg/Tag für 3 bis 5 Tage oder 100–250 mg/Monat
	Beriberi: Neuropathie, Schmerzen, Parästhesie, Reflexverlust, Herzinsuffizienz, Ödeme			
Vitamin B_12_	Makrozytäre (megaloblastäre) Anämie, milde Panzytopenie, neuropsychiatrische Symptome: periphere Neuropathie mit gestörter Propriozeption, axonale oder optische Neuropathie, Depression, verlangsamtes Denken	2,4 µg	350–1000 µg/Tag p.o. oder 1000 µg/Monat i.m./s.c. oder als Nasenspray	1000 µg/Tag bis zur Normalisierung der Serumspiegels, dann Wiederaufnahme der Erhaltungsdosis
Folat	Makrozytäre (megaloblastäre) Anämie, milde Panzytopenie, prädominant sensorische Neuropathie, Neuralrohrdefekte	400 µg	Allgemein: 400–800 µg/Tag als Multivitaminpräparat	1000 µg/Tag p.o. bis zur Normalisierung der Serumspiegel, dann Wiederaufnahme der Erhaltungsdosis
			Frauen im gebärfähigen Alter: 800–1000 µg/Tag; maximale Tagesdosis: 1 mg	
Eisen	Anämie, Pica-Syndrom, Lernstörungen	Männer ≥ 19 Jahre und Frauen ≥ 51 Jahre: 8 mg/Tag	Männer, postmenopausale Frauen und Personen ohne Anämieanamnese: 18 mg als Multivitaminpräparat	150–300 mg p.o. 2‑ bis 3‑mal/Tag; parenterale Verabreichung, wenn kein Response auf orale Supplementation
		Frauen zwischen 19 und 50 Jahren: 18 mg/Tag	Menstruierende Frauen und Frauen/Männer nach RYGB, SG: > 45–60 mg/Tag elementares Eisen (alle Quellen)	
Zink	Retardiertes Wachstum, verzögerte sexuelle Reife, Impotenz, beeinträchtigte Immunfunktion	Frauen: 8 mg	RYGB: 8–22 mg (100–200 % RDA)	Die optimale Dosis ist nicht bekannt; Überdosierungen können toxisch wirken oder Kupferdefizienz zur Folge haben
		Männer: 11 mg	SG: 8–11 mg (100 % RDA)	
			Erhaltungsdosis: 8–15 mg Zink pro 1 mg Kupfer	
Kupfer	Anämie, Neutropenie, Ataxie	900 µg	RYGB: 2 mg/Tag (200 % RDA);	Milde bis moderate Defizienz: 3–8 mg Kupfer p.o. bis zur Normalisierung der Serumspiegel
			SG: 1 mg/Tag (100 % RDA)	Schwere Defizienz: 2–4 mg i.v. für 6 Tage oder bis zum Abklingen der Symptome
			Erhaltungsdosis: 8–15 mg Zink pro 1 mg Kupfer	
Selen	Skelettomuskuläre Dysfunktion und Kardiomyopathie, Stimmungsschwankungen, beeinträchtigte Immunfunktion, Makrozytose	55 µg	Nicht bekannt, aber vermutlich > 100 µg/Tag	2 µg/kg/Tag bei Entwicklung einer Kardiomyopathie
Kalzium	Knochenerkrankungen, sekundärer Hyperparathyreoidismus	1000–1200 mg	RYGB, SG: 1200–1500 mg/Tag (in mehreren Dosen)	RYGB, SG: 1200–1500 mg/Tag (in mehreren Dosen)

*RYGB* Roux-en-Y-Magenbypass, *SG* „sleeve gastrectomy“

Eine Laborkontrolle der relevanten Mikronährstoffe wird im ersten Jahr nach bariatrischer Operation nach 1, 3, 6 und 12 Monaten, im zweiten postoperativen Jahr halbjährlich sowie anschließend jährlich empfohlen. Im Rahmen dieser Kontrollen sollte stets auch ein Screening auf mögliche Symptome eines Nährstoffmangels erfolgen. Bei allen PatientInnen wird nach bariatrischer Operation eine Supplementierung von Mikronährstoffen empfohlen, bei laborchemisch nachgewiesenem Mangel oder typischer Symptomatik eines Mangels sollte eine entsprechende Aufsättigung erfolgen (Tab. 2; [7, 9, 10]).

### Fettlösliche Vitamine

Die fettlöslichen Vitamine A, D, E und K werden v. a. im Jejunum und Ileum resorbiert. Insbesondere nach Roux-en-Y-Magenbypass (RYGB) ist mit einem Mangel fettlöslicher Vitamine zu rechnen [11]. Aufgrund dieser häufigen Mängel wird eine Supplementierung fettlöslicher Vitamine bei diesen Operationsverfahren empfohlen (Tab. 2):

*Vitamin A***. **Ein Vitamin-A-Mangel ist laut Literatur bei 6–14 % aller PatientInnen mit Adipositas zu finden [7, 12]. Nach SG und RYGB entwickeln bis zu 13 % einen Mangel [5]. Nachtblindheit, Wundheilungsstörungen, Geschmacksveränderungen und Hyperkeratosen sind mögliche frühe Anzeichen eines Vitamin-A-Mangels, bei andauerndem Mangel kann es zu Schäden der Hornhaut bis hin zur Erblindung kommen. Die empfohlene Supplementierung beträgt 5000–10.000 IU/Tag nach RYGB und SG. Höhere Erhaltungsdosen können erforderlich sein. Für PatientInnen mit Vitamin-A-Mangel ohne Hornhautveränderungen wird Vitamin A oral in einer Dosis von 10.000–25.000 IE/Tag empfohlen, bis klinische Besserung eintritt. Bei vorliegendem Vitamin-A-Mangel und Hornhautveränderungen sollte eine Dosis von 50.000–100.000 IE Vitamin A für 3 Tage i.m. verabreicht werden, gefolgt von 50.000 IE/Tag i.m. für 2 Wochen. Ein zusätzlich vorliegender Eisen- und/oder Kupfermangel kann die Behebung des Vitamin-A-Mangels erschweren [9].

*Vitamin D***. **In Studien wurde ein Vitamin-D-Mangel bereits präoperativ in sehr hohem Ausmaß diagnostiziert [12], nach bariatrischer Operation konnte bei nahezu 100 % der PatientInnen ein Defizit festgestellt werden [7]. Initiale Veränderungen von Kalzium- und Phosphathaushalt können in weiterer Folge unter anderem Pathologien des Knochenstoffwechsels bedingen. Die optimalen Vitamin-D-Spiegel werden teils kontrovers diskutiert, jedenfalls sollte mit einer Gesamtzufuhr von 3000 IE Vitamin D_3_ täglich ein 25(OH)D-Spiegel von > 30 ng/ml (75 nmol/l) angestrebt werden. Bei einem Mangel von Vitamin D werden entsprechend höhere Dosen zur Aufsättigung empfohlen [13–15].

*Vitamin E***. **Seltener als bei anderen fettlöslichen Vitaminen findet sich ein Mangel an Vitamin E bei Adipositas (Prävalenz ca. 2 %) oder nach bariatrischer Operation [7]. Ein Vitamin-E-Mangel kann sich in Hämolyse und neuromuskulären Pathologien äußern, bei einem Defizit wird die tägliche Gabe von 90–300 mg (100–400 IU) empfohlen, die Supplementierung erfolgt mit 15 mg (22,4 IU) [7, 9, 10].

*Vitamin K*. Vitamin-K-Mangel führt zu einer beeinträchtigten Blutgerinnung mit entsprechender Klinik und möglicher Blutungsneigung. Die Empfehlung zur Supplementierung umfasst 90–120 µg Vitamin K nach RYGB oder SG. Bei akuter oder chronischer Malabsorption werden entsprechend höhere Dosierungen benötigt [7, 9].

### Wasserlösliche Vitamine

Die Resorption von Kalzium, Eisen, Zink und Kupfer erfolgt insbesondere durch Transporter im Duodenum und proximalen Jejunum. Bei bariatrischen Operationsverfahren wie bei RYGB ist durch die Ausschaltung dieser Darmabschnitte ein Mangel der entsprechenden Nährstoffe häufig. Somit wird eine regelhafte Supplementierung empfohlen (Tab. 2):

*Vitamin B*_*1*_* (Thiamin)***. **Ein Vitamin‑B_1_-Mangel kann je nach Operationsverfahren bei bis zur Hälfte der PatientInnen nach bariatrischer Operation auftreten [7]. Insbesondere nach RYGB wird die Aufnahme im Duodenum und proximalen Jejunum stark reduziert. Bei anhaltender Übelkeit mit Erbrechen sollte jedenfalls eine neurologische Abklärung erfolgen und an einen Vitamin‑B_1_-Mangel gedacht werden. Bei einem ausgeprägten Mangel kann sich eine neurologische Symptomatik mit Enzephalopathie, Ataxie und Okulomotorius-Dysfunktion (Wernicke-Enzephalopathie) entwickeln [16–18]. Daher wird eine Prophylaxe mit mindestens 12 mg Thiamin täglich empfohlen, am besten in Form von ausreichend dosierten Vitamin-B-Komplex-Präparaten. Für die Gabe von Thiamin bei (symptomatischen) Defiziten gibt es Empfehlungen für eine orale, intravenöse oder intramuskuläre Supplementierung [9].

*Vitamin B*_*12*_**. **Während präoperativ die Prävalenz eines Vitamin‑B_12_-Mangels bereits mit bis zu 18 % angegeben wird [7], steigt diese je nach Operationsmethode auf bis zu 30 % und mehr nach RYGB [18]. Die klassische Folge eines Vitamin‑B_12_-Mangels ist eine hyperchrome makrozytäre (perniziöse) Anämie. Eine periphere Neuropathie kann ebenfalls aus einem Vitamin‑B_12_-Mangel resultieren. Postoperativ wird eine orale Supplementierung von 350–1000 µg pro Tag empfohlen. Als Alternative steht die Gabe von 1000 µg Vitamin B_12_ subkutan oder intramuskulär 1‑mal pro Monat zur Verfügung [19]. Das Dosierungsintervall sollte im Verlauf nach Bedarf angepasst werden.

*Folsäure *wird im gesamten Dünndarm resorbiert. Wie auch beim Vitamin‑B_12_-Mangel kann ein Folsäuremangel zu einer hyperchromen makrozytären Anämie führen. Anders als beim Vitamin‑B_12_-Mangel bestehen jedoch im Körper keine Speicher für mehrere Monate, daher kann auch eine insuffiziente Folsäurezufuhr rasch zu einem Mangel führen. Die empfohlene Supplementierung erfolgt mit 400–800 µg (bei Frauen im gebärfähigen Alter 800–1000 µg) täglich, meist in Form von Kombinationspräparaten. Ein dokumentierter Folsäuremangel wird mit 1000 µg pro Tag bis zur Normalisierung des Folsäurewertes substituiert [9, 18].

*Vitamin C***. **Bei Einhaltung der Ernährungsempfehlungen mit ausreichender Zufuhr von Obst und Gemüse ist ein Vitamin-C-Mangel postoperativ selten. Die Gabe eines Vitamin-C-Präparates kann bei insuffizientem Ansprechen auf eine orale Eisenzufuhr erwogen werden, um die Resorption von Eisen zu verbessern [9].

### Spurenelemente

*Eisen***. **Das typische Zeichen eines Eisenmangels ist der Nachweis einer mikrozytären hypochromen Anämie. Eisenmangel stellt die häufigste Mangelerscheinung dar und findet sich postoperativ bei bis zu 50 %. Die Eisenaufnahme erfolgt hauptsächlich im Duodenum und proximalen Jejunum, somit ist insbesondere nach RYGB mit einem Mangel zu rechnen. Frauen haben durch die Menstruation ein erhöhtes Risiko, einen Eisenmangel zu entwickeln. Daher wird bei der Substitution bei Männern und fehlender Anämieanamnese die Gabe von 18 mg pro Tag in Form eines Multivitaminpräparates und bei menstruierenden Frauen und nach RYGB sowie nach SG eine höhere Dosis von mindestens 45–60 mg pro Tag empfohlen. In der täglichen Praxis ist eine orale Eisensubstitution oftmals insuffizient. Insbesondere bei ausgeprägt niedrigen Ferritinwerten und bereits symptomatischer Anämie ist eine intravenöse Eisengabe oftmals zielführender [9, 20].

*Kupfer* wird für eine normale Nervenfunktion sowie bei der Hämatopoese benötigt und wird im Magen und proximalen Duodenum resorbiert [21]. Ein Kupfermangel kann zu einer mikrozytären Anämie, Neutropenie und Ataxie führen. Je nach Operationsverfahren wird eine postoperative Supplementierung von 2 mg Kupfer bei RYGB und von 1 mg Kupfer bei SG empfohlen. Des Weiteren wird bei Zinkeinnahme pro 8–15 mg Zink die Supplementierung von 1 mg Kupfer empfohlen, um einem Kupfermangel vorzubeugen. Bei einem laborchemischen Kupfermangel wird die Substitution von 3–8 mg Kupfer oral pro Tag empfohlen, bei neurologischen Symptomen ist die Gabe von 2–4 mg Kupfer intravenös für 6 Tage oder für die Dauer der Beschwerden indiziert [9].

*Zink.* Ein Zinkmangel kann sich unter anderem mit Infektanfälligkeit, Dermatitis und Impotenz manifestieren. Die Zinkaufnahme erfolgt großteils im Duodenum und proximalen Jejunum. Die Wahrscheinlichkeit eines Zinkmangels schwankt in Studien stark und liegt bei bis zu 40 % nach RYGB. Die empfohlene postoperative Supplementierung mit einem Multivitaminpräparat richtet sich nach der Operationsmethode (RYGB: 8–22 mg/Tag, SG: 8–11 mg/Tag). Die optimale Dosis bei nachgewiesenem Zinkmangel ist unbekannt [7].

*Selen. *Ein Selenmangel wird mit Kardiomyopathien, motorischen Funktionseinschränkungen, psychischen Beeinträchtigungen und Infektneigung assoziiert. Die Resorption von Selen findet im Duodenum und proximalen Jejunum statt. Postoperativ ist bei bis zu 15 % der PatientInnen mit einem Selenmangel zu rechnen [21]. Eine Supplementierung mit zumindest 50 µg pro Tag wird empfohlen, wobei die optimale Dosis unbekannt ist und vermutlich > 100 µg pro Tag notwendig sind. In Studien wurde bei einem Selenmangel mit Kardiomyopathie eine Substitution mit 2 µg/kg/Tag durchgeführt [22].

*Kalzium.* PatientInnen mit einem Kalziummangel können neben einem gestörten Knochenstoffwechsel auch einen sekundären Hyperparathyreoidismus entwickeln. Die Absorption von Kalzium ist beeinflusst durch Vitamin D und findet bevorzugt in einem sauren Milieu im Duodenum und proximalen Jejunum statt. Postoperativ können daher eine geringere Kalziumzufuhr, ein Vitamin-D-Mangel und der Mangel an Magensäure zu einem Kalziummangel führen. Eine postoperative Supplementierung von 1200–1500 mg/Tag bei RYGB und SG wird empfohlen [9].

## Versorgung mit Makronährstoffen

Bei den Makronährstoffen gilt es v. a., auf eine ausreichende Proteinversorgung zu achten, um den Verlust fettfreier Körpermasse vorzubeugen – insbesondere in den ersten postoperativen Monaten. Dabei sollte eine minimale Proteinzufuhr von 60 g/Tag und bis zu 1,5 g/kg ideales Körpergewicht pro Tag angestrebt werden. Der genaue Bedarf muss individuell ermittelt werden. Proteinsupplemente können zur Erreichung der benötigten Proteinmenge zu Hilfe genommen werden [23].

## Typ-2-Diabetes

Typ-2-Diabetes (T2D) ist eine häufige Begleiterkrankung von Adipositas, welche einen der Haupttreiber der T2D-Pandemie darstellt. Die bariatrische Chirurgie führt nachweislich zur Verbesserung des metabolischen Profils bis zur Remission des T2D sowie zur Reduktion mikro- und makrovaskulärer Komplikationen und kardiovaskulärer Todesfälle bei PatientInnen mit T2D [24–27].

In einer randomisierten klinischen Studie erreichten PatientInnen nach SG oder RYGB im Vergleich zu medikamentös behandelten PatientInnen neben einer überlegenen Gewichtsreduktion auch eine signifikant bessere HbA_1c_-Reduktion von 2,1 %-Punkten gegenüber dem Ausgangswert. Die Effekte auf die Blutzuckerkontrolle waren dauerhaft und auch bei initial leichter Adipositas mit einem Body Mass Index (BMI) von 27–34 kg/m^2^ zu beobachten [26]. Prädiktoren für das Erreichen einer Diabetesremission waren eine kürzere Diabetesdauer und die Durchführung eines Magenbypass als bariatrisches Operationsverfahren [26, 27], während ein weiter fortgeschrittener T2D mit längerer Diabetesdauer, höherem HbA_1c_, höhere Anzahl an oraler antidiabetischer Therapie oder Insulinbedarf sowie höheres Alter eher gegen eine Remission sprachen [28]. Aus Daten der Swedish Obese Subjects(SOS)-Studie ging hervor, dass 72 % der PatientInnen mit T2D 2 Jahre nach bariatrischer Operation in Remission waren [24]. Die metabolischen Effekte der bariatrischen Chirurgie beruhen nicht nur auf der Nahrungsrestriktion und der Gewichtsabnahme, sondern unter anderem auch auf der anatomischen Veränderung des Magen-Darm-Trakts mit hormonellen Veränderungen, wie z. B. höheren GLP-1-Konzentrationen [29].

T2D ist eine progrediente Erkrankung mit fortschreitendem Verlust der Betazellfunktion. Initial reicht zur glykämischen Kontrolle eine Pharmakotherapie, welche im Verlauf womöglich bis zur Insulinbehandlung eskaliert werden muss. Eine Gewichtsabnahme von 10–15 % reicht in frühen T2D-Stadien für eine Remission aus. Beim Erreichen der Diabetesremission spielen die Umkehrung der ektopischen Lipidablagerung in Leber, Pankreas und Muskeln sowie Wiederherstellung der Insulinsensitivität und der Betazellfunktion eine wesentliche Rolle [30]. In einem frühen T2D-Stadium steigt die Wahrscheinlichkeit einer Remission nach einer metabolischen Operation ab einer Gewichtsabnahme von 10–15 %. Bei einem fortgeschrittenen T2D, insbesondere bei etablierter Insulintherapie, scheint eine Gewichtsabnahme von 20–25 % für das Erreichen einer Remission erforderlich [31]. Obwohl die Effekte der bariatrischen Chirurgie dauerhaft sind und auch die Verbesserung der glykämischen Kontrolle in der Regel länger andauert, kann es durch eine neuerliche Gewichtszunahme und den fortschreitenden Verlust der Betazellfunktion im Laufe der Zeit wieder zu einem Blutzuckeranstieg kommen [29].

### Peri- und unmittelbar postoperatives Diabetesmanagement

Die bariatrische Chirurgie führt unmittelbar postoperativ zur Verbesserung der glykämischen Kontrolle. Daher ist eine rasche Anpassung der medikamentösen Therapie notwendig. Präoperativ sollte daran gedacht werden orale Antidiabetika und GLP-1-Rezeptoragonisten rechtzeitig vor der Operation zu pausieren. Die Basalinsulindosis sollte auf ≤ 0,3 IE/kg reduziert werden. Im Krankenhaus sollten die Zielglukosewerte 140–180 mg/dl betragen [1].

Postoperativ sollten Medikamente, die das Risiko einer Hypoglykämie erhöhen, wie beispielsweise Sulfonylharnstoffe, vermieden werden. Metformin kann bei Bedarf ab dem dritten postoperativen Tag wieder begonnen werden, wobei zuvor die Nierenfunktion kontrolliert werden sollte. Nach RYGB ist die biologische Verfügbarkeit von Metformin um 50 % erhöht, sodass eine Dosis von 850 mg 2‑mal täglich ausreichend ist. Am Operationstag sollten kurz wirksame Insulinanaloga verwendet werden, um eine Hyperglykämie zu korrigieren [1]. Bei komplikationslosem Verlauf kann am ersten postoperativen Tag ein Wiederbeginn mit Basalinsulin erwogen werden, falls die Fortführung der Basalinsulintherapie notwendig erscheint. Dabei werden Basalinsulindosen zwischen 0,1 IE/kg [1] und der Hälfte der präoperativen Basalinsulindosis [32] empfohlen. Engmaschige Blutzuckerkontrollen müssen erfolgen. Bei PatientInnen mit postoperativ anhaltendem Insulinbedarf sollte die Insulindosis in Rücksprache mit einer/m FachärztIn für Endokrinologie und Diabetologie angepasst und engmaschige Kontrollen vereinbart werden [1]. Vor dem Absetzen der Insulintherapie sollte daran gedacht werden, dass auch bei Menschen mit Adipositas ein Autoimmundiabetes und ein damit einhergehender Insulinbedarf vorliegen könnten.

### Definition einer Diabetesremission

Eine Diabetesremission wird definiert als eine Rückkehr des HbA_1c_ auf < 6,5 %, gemessen mindestens 3 Monate nach dem bariatrischen Eingriff und 3 Monate nach dem Absetzen der glukosesenkenden Pharmakotherapie. Als alternative Marker kann ein Nüchternblutzucker < 126 mg/dl oder ein GMI („glucose management indicator“) < 6,5 %, berechnet aus CGM-Werten, herangezogen werden.

Zur Feststellung einer Diabetesremission sollte der HbA_1c_ unmittelbar vor dem bariatrischen Eingriff und frühestens 3 Monate postoperativ bzw. 3 Monate nach dem Absetzen der glukosesenkenden Pharmakotherapie gemessen werden [29].

### Monitoring

Trotz initial hoher Diabetesremissionsraten muss bedacht werden, dass es über die Jahre aufgrund einer neuerlichen Gewichtszunahme und/oder eines fortschreitenden Betazellfunktionsverlusts wieder zu einem Diabetesrückfall kommen kann. Von den SOS-PatientInnen, die nach 2 Jahren eine Diabetesremission aufwiesen, hatten 50 % nach 10 Jahren eine Diabetesrückfall [24]. Daher muss nach eingetretener Diabetesremission ein Monitoring erfolgen, um eine wiederauftretende Hyperglykämie frühzeitig zu erkennen. Der HbA_1c_-Wert oder ein anderes Maß für die Kontrolle des Blutzuckerspiegels sollte mindestens 1‑mal jährlich gemessen werden. Die Aufrechterhaltung eines gesunden Lebensstils muss kontinuierlich überwacht werden. Eine medikamentöse Therapie für andere Erkrankungen, die bekanntermaßen mit einer Hyperglykämie einhergehen, wie beispielsweise Glukokortikoide oder bestimmte Antipsychotika, sollte nach Möglichkeit vermieden werden [29].

Der „legacy effect“, auch als „metabolisches Gedächtnis“ bezeichnet, beschreibt die langfristigen schädlichen Auswirkungen einer früheren Hyperglykämie in verschiedenen Geweben. Als mögliche Ursachen werden unter anderem die Produktion reaktiver Spezies, die Glykierung mitochondrialer Proteine und die Induktion einer veränderten Genexpression diskutiert [33]. Diese Veränderungen bleiben auch bestehen, wenn der Blutzucker normalisiert ist. Daher ist einerseits eine frühzeitige intensive Blutzuckeroptimierung bei wiederkehrender Hyperglykämie nach Remission essenziell, um das Risiko diabetischer mikro- und makrovaskulärer Komplikationen und Ereignisse zu reduzieren, andererseits muss auch nach eingetretener Diabetesremission bei Normoglykämie ein Screening auf Diabetes-assoziierte Folgeerkrankungen erfolgen [29].

Auch bei Vorliegen einer Diabetesremission sollte jährlich auf diabetesspezifische Folgeerkrankungen gescreent werden:

- augenärztliche Kontrolle inklusive Netzhautuntersuchung zum Ausschluss einer diabetischen Retinopathie
- Bestimmung von Kreatinin, GFR, Albumin-Kreatinin-Ratio zum Ausschluss einer diabetischen Nephropathie
- Fußuntersuchung
- Blutdruckmessung
- Screening auf diabetische Neuropathie (Mikrofilamenttest, Stimmgabeltest)

Bei Vorliegen einer Retinopathie kann es nach bariatrischer Chirurgie, insbesondere bei initial unzureichend kontrollierter Stoffwechsellage, zu einer abrupten Verschlechterung durch eine rasche Senkung des Blutzuckerspiegels kommen. In diesem Fall sollte ein engmaschiges Netzhautscreening erfolgen.

## Dumping-Syndrom

Unter „Dumping“ versteht man das postprandiale Auftreten einer Konstellation von Symptomen, die durch den schnellen Übergang von kalorienreicher Nahrung in den Dünndarm ausgelöst wird [9, 34, 35]. Es ist ein häufiger Befund bei PatientInnen nach bariatrischen Operationen [36]. Trotz der auffälligen Symptome, die unspezifisch sein können, ist eine korrekte Diagnose nicht immer einfach. Im Allgemeinen unterscheidet man zwischen Typ-1- und Typ-2-Dumping. Beide Typen treten häufig in Kombination auf, da beide durch die schnelle Nahrungsaufnahme ausgelöst werden.

*Typ-1-Dumping*, früher als Early Dumping bezeichnet, tritt typischerweise innerhalb der ersten Stunde nach einer Mahlzeit auf (häufig 10–30 min nach der Nahrungsaufnahme) und ist mit gastrointestinalen (Bauchschmerzen, Blähungen, Übelkeit, Durchfall, Borborygmus) und vasomotorischen Symptomen (Flushing, Schwitzen, Hypotonie, Müdigkeit, Wunsch, sich hinzulegen, Herzklopfen, Tachykardie, sehr selten Synkope) verbunden [37, 38]. Das Dumping-Syndrom vom Typ 1 wird auf die rasche Entleerung des hyperosmolaren Speisebreis in den Darm zurückgeführt, was einen Flüssigkeitseinstrom in das Darmlumen, eine Darmdistension, eine Flüssigkeitssequestrierung, ein verringertes intravaskuläres Volumen und eine Hypotonie zur Folge hat [37].

*Typ-2-Dumping*, früher auch als Spätdumping bezeichnet, tritt 1–3 h nach der Nahrungsaufnahme auf [38]. Die reaktive Hypoglykämie folgt einer Hyperinsulinämie, die durch Veränderungen der gastrointestinalen Hormone (z. B. Anstieg von GLP-1) und der Insulinsekretion bedingt ist, welche nach bariatrischen Eingriffen beschrieben wurden [34, 39, 40]. Die einhergehenden Symptome, angefangen von neuroglykopenischen (Müdigkeit, Schwäche, Schwindel, Verwirrung, sehr selten Krampfanfälle und Synkopen) bis hin zu autonomen/adrenergen Symptomen (Hunger, Übelkeit, Herzklopfen, Tachykardie, Zittern, Schwitzen, Angst), sind mit der reaktiven Hypoglykämie assoziiert.

### Diagnose des Dumping-Syndroms

Zur Diagnose des Dumping-Syndroms kann in Anlehnung an andere Leitlinien ein postprandialer Spontanblutzucker < 50 mg/dl (2,8 mmol/l) als Cut-off-Wert verwendet werden [36]. Es gibt jedoch viele Diskussionen darüber, ob dieser Wert zu streng ist. Bei Provokationstests, wie dem oralen Glukosetoleranztest (oGTT), wird ein Anstieg des Hämatokrits um mehr als 3 % oder ein Anstieg der Herzfrequenz um mehr als 10 Schläge pro Minute in den ersten 30 Minuten nach Testbeginn als Typ-1-Dumping, und eine Hypoglykämie (< 50 mg/dl) 60–180 Minuten nach Testbeginn als Typ-2-Dumping gewertet [34, 41].

Eine korrekte Diagnose wird durch die Anwendung des Sigstad-Scores erleichtert. Das Diagnoseinstrument basiert auf der Gewichtung von Faktoren, die den Symptomen des Syndroms zugeordnet werden: Ein Score-Index von mehr als 7 Punkten deutet auf ein Typ-2-Dumping hin (Tab. 3; [42]). Dieses Scoring-System wurde jedoch in den 1970er-Jahren entwickelt und ist möglicherweise nicht immer so genau wie nötig [43], kann aber bei einem Glukosetoleranztest oder einem Mischkosttoleranztest angewendet werden.

**Table 3:** 

Symptom	Punktezahl
Schock	+5
Bewusstlosigkeit, Ohnmacht	+4
Bedürfnis, sich hinzulegen	+4
Atemlosigkeit, Dyspnoe	+3
Schwäche, Erschöpfung	+3
Schläfrigkeit, Benommenheit, Apathie, Einschlafen	+3
Palpitation	+3
Ruhelosigkeit	+2
Schwindel	+2
Kopfschmerzen	+1
Wärmeempfinden, Schwitzen, Blässe, feuchte Haut	+1
Nausea	+1
Völlegefühl, Meteorismus	+1
Borborygmus	+1
Aufstoßen	−1
Erbrechen	−4

**Table 4:** 

	Jahr 1^a^	Jahr 2^a^	Folgejahre
**Anamnese**			
Beschwerden erfragen (Reflux, Dumping, Suchtverlagerung etc.)	6, 12	18, 24	Jährlich
Regelmäßige Einnahme der Supplemente erfragen und bestärken	6, 12	18, 24	Jährlich
Medikamente überprüfen	6, 12	18, 24	Jährlich
Gewichtsverlauf dokumentieren (wenn nicht bekannt Gewicht präoperativ, tiefstes Gewicht postoperativ etc. erfragen)	6, 12	18, 24	Jährlich
**Monitoring von Ernährungsverhalten und -therapie**			
Ernährungsprotokoll (inklusive Beschwerden) und Ernährungsberatung	1, 3, 6, 12	18, 24	Jährlich
Auf Proteinaufnahme achten (Supplementierung)	1, 3. 6, 12	18, 24	Jährlich
Bei Dumping-Syndrom diätologische Intervention als erste Therapiewahl	1, 3, 6, 12	18, 24	Jährlich
Ärztliche Zuweisung zur Diätologie (im niedergelassenen Bereich zur Kostenübernahme)	1, 3, 6, 12	18, 24	Jährlich
**Bewegungsempfehlungen**			
Auf allgemeine Bewegungsempfehlungen hinweisen (150 min/Woche mittlere Intensität oder 75 min/Woche höhere Intensität plus Krafttraining [2 bis 3 Einheiten/Woche])	6, 12	18, 24	Jährlich
**Psychologische Betreuung**			
Idealerweise psychologische Langzeitbetreuung	6, 12	18, 24	Jährlich
Psychologische Risiken evaluieren:	6, 12	18, 24	Jährlich
– Suizidalität			
– Substanzabhängigkeit			
– Essstörungen			
**Laborkontrollen (Minimalanforderungen)**			
*Für alle bariatrischen Eingriffe*			
Blutbild, Elektrolyte (Kalzium, Phosphat), Eisenstatus (Eisen, Ferritin), Vitamin B_12_, Folsäure, 25-OH-Vitamin D, PTH, Albumin, Leberwerte, Nierenwerte	1, 3, 6, 12	18, 24	Jährlich
Harneiweißausscheidung (Albumin/Kreatinin-Ratio)	1, 3, 6, 12	18, 24	Jährlich
*Für malabsorptivere Verfahren (Magenbypass)*			
Kalzium im 24-h-Harn	1, 3, 6, 12	18, 24	Jährlich
β‑CrossLaps, Osteocalcin	1, 3, 6, 12	18, 24	Jährlich
Vitamin A, E	1, 3, 6, 12	18, 24	Jährlich
Kupfer, Selen, Zink, Magnesium	1, 3, 6, 12	18, 24	Jährlich
Gerinnung (aPTT, PTZ, INR)	1, 3, 6, 12	18, 24	Jährlich
**Supplementation**			
Anpassung der Supplementation, basierend auf den Laborergebnissen	1, 3, 6, 12	18, 24	Jährlich
Eignung des Multivitaminpräparat für jeweiligen bariatrischen Eingriff evaluieren	1, 3, 6, 12	18, 24	Jährlich
Zusätzlich zum Multivitaminpräparat nötige Supplementierung (Protein, Vitamin B_12_, Eisen, Kalzium, Vitamin D)	1, 3, 6, 12	18, 24	Jährlich
**Spezifische Untersuchungen**			
Knochendichtemessung	–	24	Alle 2 Jahre
Gastroskopie (nach Sleeve-Gastrektomie)		–	Nach 3 Jahren, dann alle 5 Jahre

^a^ Visiten in Monaten postoperativ

Eine weitere Möglichkeit wäre das Arts-Scoring-System. Es unterscheidet zwischen Typ-1- und Typ-2-Dumping, indem es Symptome verwendet, die entweder mit frühem oder spätem Dumping verbunden sind. Diese Symptome sollten dann auf einer vierstufigen Likert-Skala bewertet werden (fehlend bis schwer) [34].

Eine alternative Methode, die immer mehr an Bedeutung gewinnt, ist die Verwendung von kontinuierlichen Glukosemesssystemen (CGM) für die Diagnose [44].

Das Dumping-Syndrom tritt nach Magenbypass (70–75 % der PatientInnen im ersten Jahr nach der Operation) [9], aber auch nach SG (40 % der PatientInnen nach 6 Monaten) auf [45]. Einige Autoren vermuteten, dass das Dumping-Syndrom durch die negative Konditionierung auf den Verzehr von Nahrungsmitteln mit hohem Energiegehalt bei der Gewichtsabnahme nach einem Magenbypass eine Rolle spielen könnte [46], wobei diese Rolle infrage gestellt wurde [43].

### Therapie des Dumping-Syndroms

Eine Ernährungsumstellung ist in vielen Fällen ausreichend, um das Dumping-Syndrom zu kontrollieren. Die Ernährungstipps umfassen kleine, aber häufige Mahlzeiten, die Vermeidung von Flüssigkeitszufuhr innerhalb von 30 min nach einer festen Mahlzeit, die Vermeidung von Einfachzucker, die Erhöhung der Aufnahme von Ballaststoffen und komplexen Kohlenhydraten sowie die Erhöhung der Proteinzufuhr [9].

Eine mögliche Ergänzung könnte die Verwendung von Nahrungsergänzungsmitteln sein, die die Viskosität der Nahrung erhöhen. Die Hypothese ist, dass eine erhöhte Konsistenz der Nahrung die Magenentleerung verlangsamt und somit die Geschwindigkeit, mit der die Nährstoffe im Dünndarm freigesetzt werden, verringert [34]. Es wurden mehrere Studien mit Guarkernmehl oder Pektin durchgeführt. Allerdings war die Zahl der StudienteilnehmerInnen zu gering, um eine endgültige Entscheidung über den Einsatz dieser Nahrungsergänzungsmittel zu treffen [37–49].

Eine medikamentöse Therapie kommt bei PatientInnen zum Einsatz, bei denen diätetische Maßnahmen nicht ausreichen, um die Krankheit unter Kontrolle zu bringen. Mehrere Medikamente wurden als vorteilhaft für die Kontrolle der Symptome beschrieben, jedoch ohne durchgängigen Erfolg.

*Acarbose* kann und sollte als Erstlinienmedikament zur Behandlung von Typ-2-Dumping eingesetzt werden, da es eine bekannte und sichere Option ist. Aufgrund der gastrointestinalen Nebenwirkungen wird Acarbose jedoch häufig von den PatientInnen nicht gut vertragen, insbesondere nach bariatrischen Operationen [50–52].

*Octreotid.* Die Wirksamkeit von Octreotid in einer empfohlenen Anfangsdosis von 25–50 µg, die 15–30 min vor den Hauptmahlzeiten subkutan verabreicht wird, bei der Linderung von Typ-1- als auch Typ-2-Dumping-Symptomen wird durch mehrere randomisierte Studien belegt [53]. Dieses Medikament kann daher bei einigen PatientInnen hilfreich sein [9, 34].

*Weitere Optionen.* Diazoxid reduziert die Kalzium-induzierte Insulinfreisetzung. Die Verwendung zur Behandlung des Dumping-Syndroms ist nur in Fallserien beschrieben [54]. Auch der Einsatz von Liraglutid ist bisher nur in Fallberichten beschrieben und kann daher nicht generell empfohlen werden [55]. Ein weiterer vielversprechender Therapieansatz könnte der Einsatz von SGLT2-Inhibitoren sein. Kleine Pilotstudien mit Empagliflozin und Canagliflozin zeigten positive Effekte [56, 57]. Es sind jedoch weitere Studien erforderlich, um diese positiven Wirkungen genauer zu bewerten.

Bei schweren hypoglykämischen Ereignissen mit neuroglykopenischen Symptomen sollten jedoch alternative Ursachen der hyperinsulinämischen Hypoglykämie (Hyperplasie der Betazellen des Pankreas, Insulinom) in Betracht gezogen werden. Dafür wurden spezifische Diagnosealgorithmen vorgeschlagen [58].

Bleiben alle Bemühungen ohne ausreichenden Erfolg, kann eine erneute chirurgische Intervention in Erwägung gezogen werden. Nach einem Roux-en-Y-Magenbypass (RYGB) sind die häufigsten Eingriffe die Umkehrung des Bypasses oder die Verkleinerung des Pouches [34, 59, 60]. Allerdings handelt es sich bei keiner dieser Studien um randomisierte Versuche. In einer Metaanalyse mit 14 Studien und 75 PatientInnen wurde beschrieben, dass ein Teil der PatientInnen von der chirurgischen Re-Intervention profitiert, jedoch wurden im Verlauf eine Gewichtszunahme, Diabetes und wiederkehrende Hypoglykämien beschrieben [61]. Eine partielle oder sogar totale Pankreatektomie sollte nur als Ultima Ratio eingesetzt werden [62].

## Internistische Komplikationen nach bariatrischen Operationen

Ganz allgemein kann in frühe Komplikationen (bis zu 30 Tage postoperativ) und späte Komplikationen unterteilt werden.

### Frühe Komplikationen

Zu den häufigsten frühen Komplikationen zählen Insuffizienzen der Anastomosen, Blutungskomplikationen (bis zu 6 %) und thromboembolische Ereignisse. Die Komplikationen treten je nach Operationsmethode in unterschiedlicher Häufigkeit auf. Bei RYGB treten Stenosen an den Nahtstellen mit einer Frequenz von etwa 3 % auf, bei 3,1 % kommt es zu Blutungskomplikationen, Reoperationen sind bei etwa 4 % der PatientInnen erforderlich. Bei SG treten Blutungskomplikationen bei etwa 2 % der PatientInnen auf, Reoperationen sind bei 3,5 % der PatientInnen erforderlich [63]. Akute Beschwerden bedürfen einer umgehenden Abklärung, welche immer interdisziplinär durchgeführt werden sollte.

### Späte Komplikationen

Cholezystolithiasis, Pankreatitis, Anastomosenulzera (5 %), Perforationen (2 %), Blutungen aus Ulzera (5 %) und innere Hernien [64] sind typische späte Komplikationen. Je nach Kollektiv liegt die Inzidenz von Gallensteinen nach bariatrischer Chirurgie bei 122,2 pro 10.000 Personenjahre verglichen mit 22,2 pro 10.000 Personenjahren ohne bariatrische Operation. Insgesamt kommt es zu einem 5,4fach erhöhten Auftreten einer Cholezystektomie nach bariatrischen Operationen im Vergleich zur Normalbevölkerung [65].

Auch die Inzidenz einer akuten Pankreatitis nach bariatrischen Operationen ist mit 1,04 % innerhalb von 3,5 Jahren erhöht; besondere Risikofaktoren sind schneller Gewichtsverlust und intraoperative Komplikationen [66].

Gastroösophagealer Reflux und Ulzerationen treten besonders nach SG gehäuft auf (20–30 %). Neben einer entsprechenden Modifikation der Ernährung sollte frühzeitig eine Endoskopie durchgeführt werden, und generell sollten, wenn möglich, nichtsteroidale Antirheumatika gemieden werden. Esomeprazol sollte aufgrund der effektiveren Wirksamkeit und der Löslichkeit bevorzugt verabreicht werden, bei der Verordnung muss aber das höhere Interaktionsrisiko mit anderen Medikamenten über CYP2C19 beachtet werden.

### Bauchschmerzen

Bauchschmerzen stellen ein häufiges und ernst zu nehmendes Symptom nach bariatrischen Operationen dar. Innerhalb des ersten Jahres nach einem bariatrischen Eingriff kommt es doch bei 25,6 % der PatientInnen zu einem ungeplanten ärztlichen Kontakt aufgrund von Bauchschmerzen [67].

Bauchschmerzen nach bariatrischen Operationen stellen immer ein ernst zu nehmendes Symptom dar und gehören zeitnah abgeklärt. Letztlich sind bei Erstmanifestation weitere diagnostische Schritte im Sinne einer Computertomographie und in weiterer Folge, falls notwendig, einer Gastroskopie erforderlich. Der diagnostische Prozess und die Festlegung des Behandlungsweges sollten immer gemeinsam mit der Chirurgie erfolgen, um seltene, mitunter lebensbedrohliche Komplikationen wie beispielsweise innere Hernien nicht zu übersehen.

Können akute, mitunter lebensbedrohliche Ursachen ausgeschlossen werden und persistieren die Beschwerden, so sollte unbedingt das Vorliegen eines „small intestinal bacterial overgrowth“ (SIBO) in Betracht gezogen werden. Die klinische Manifestation ist meist variabel (Blähungen, Übelkeit, Diarrhö, gelegentlich Bauchschmerzen). Zur Diagnosestellung kann ein Glukose‑H_2_-Atemtest (Spezifität 78–97 %, Sensitivität 16–62 %) oder eine jejunale Sekretaspiration durchgeführt werden. Bestätigt sich das Vorliegen eines SIBO, so sollte ein Therapieversuch mit Rifaximin durchgeführt werden [68].

### Leberversagen

Vereinzelt gibt es Fallberichte von PatientInnen mit akuten schweren Einschränkungen der Leberfunktion bis hin zu akutem Leberversagen, welches in einem Zeithorizont von Monaten bis Jahren nach der Operation auftreten kann (meist manifestiert sich das Leberversagen innerhalb weniger Monate nach der Operation). Die genauen Ursachen hierfür sind bisweilen aufgrund der sehr geringen Fallzahl nicht eindeutig geklärt. Derzeit gibt es Hinweise, dass PatientInnen mit einer bereits präoperativ vorliegenden Leberzirrhose ein etwas höheres Risiko für das akute Leberversagen nach einer bariatrischen Operation aufweisen. Weitere Risikofaktoren sind eine disproportional lange alimentäre Darmschlinge und postoperativer Eiweißmangel. Lipotoxizität als auch SIBO (welches bei bis zu 70 % der PatientInnen nach einer Operation auftreten kann) und eine kompromittierte intestinale Barriere stellen weitere mögliche Mechanismen für das Entstehen eines akuten Leberversagens dar. Die Empfehlung im Sinne eines umgehenden Beginns einer parenteralen Ernährung ist allerdings nicht evidenzbasiert [69].

### Empfehlungen zur Gastroskopie nach bariatrischer Operation

Adipositas ist ein wesentlicher Risikofaktor für die Entwicklung einer gastroösophagealen Refluxerkrankung (GERD). Wenn diese länger besteht, kann eine erosive Ösophagitis, in weiterer Folge eine Barrett-Metaplasie und in seltenen Fällen auch ein Adenokarzinom des Ösophagus entstehen [70]. Nach bariatrischer Operation, insbesondere RYGB, kann es durch den signifikanten Gewichtsverlust zur Verbesserung einer Refluxsymptomatik kommen. Es besteht jedoch auch das Risiko einer Verschlechterung bei vorbestehenden Refluxbeschwerden und sogar das Risiko eines Neuauftretens eines GERD, v. a. nach SG [71].

In der Allgemeinbevölkerung wird bei Refluxsymptomen, v. a. bei fehlender Besserung nach einem Therapieversuch mit Protonenpumpenhemmern (PPI), eine Gastroskopie empfohlen, um Komplikationen wie eine hochgradige Ösophagitis, eine Barrett-Metaplasie oder eine peptische Striktur auszuschließen [72]. In einer rezenten Metaanalyse konnte jedoch gezeigt werden, dass bei PatientInnen nach SG das Auftreten eines Barretts nicht mit Refluxsymptomen korreliert. Das Risiko für das Auftreten einer Ösophagitis stieg um 13 % pro Jahr nach SG. Die Prävalenz eines Barrett war 11,6 %, wobei die meisten Fälle 3 Jahre postoperativ entdeckt wurden. Alle berichteten Fälle waren nicht-dysplastisch [73]. Ähnliche Daten erbrachte eine österreichische Studie, welche Ergebnisse der SG nach 10 Jahren oder mehr auswertete [74]. Es fand sich ein Konversionsrate von 33 %. Dabei war in 34 % der Fälle eine Refluxerkrankung ausschlaggebend für die Konversion. Unter den restlichen nicht konvertierten PatientInnen bestand bei 57 % eine symptomatische Refluxerkrankung. Bei 16 % zeigte sich in der Gastroskopie eine Ösophagitis, bei 14 % wurde eine Barrett-Metaplasie entdeckt, wobei das Auftreten ebenfalls nicht mit den Symptomen zu korrelieren schien [74].

Daher empfehlen wir in Übereinstimmung mit den Leitlinien der American Society for Metabolic and Bariatric Surgery (ASMBS) [75], zusätzlich zu den Standardindikationen in der Allgemeinbevölkerung eine Screening-Gastroskopie unabhängig von Refluxsymptomen 3 Jahre nach SG. Falls diese Index-Gastroskopie unauffällig ist, erscheinen weitere Screening-Gastroskopien alle 5 Jahre sinnvoll.

Die European Society of Gastrointestinal Endoscopy (ESGE) definiert nur Schleimhautsegmente mit zylinderförmigem Epithel mit einer Mindestlänge von 1 cm und dem Nachweis einer spezialisierten intestinalen Metaplasie als Barrett-Metaplasie. Bei säulenförmigem Epithel, das sich weniger als 1 cm oberhalb des oberen Endes der Magenfalten erstreckt, wird die Entnahme von Zufallsbiopsien, sofern keine sichtbaren Abnormitäten vorliegen, nicht empfohlen. Gezielte Biopsien sollten in diesem Fall nur dann entnommen werden, wenn eine sichtbare Läsion vorliegt [76]. Unklar bleibt vorerst, ob das mögliche Risiko eines GERD oder Barretts nach SG höher ist als in einer Vergleichsgruppe mit Adipositas als Risikofaktor für GERD und Barrett, welche sich keiner SG unterzieht. Der Gewichtsverlust und die günstigen metabolischen Vorteile nach SG scheinen das Risiko eines GERD oder Barretts zu überwiegen.

Die Empfehlungen zum postoperativen Management nach bariatrischer Chirurgie sind in Tab. 4 zusammengefasst.

## Interessenkonflikt

J. Brix erhielt Honorare für Advisory Boards und Vorträge von Astra Zeneca, Boehringer-Ingelheim, Eli Lilly, Novo Nordisk, Sanofi. M. Resl hat von folgenden Unternehmen, die teils auch fördernde Mitglieder der ÖDG sind, Honorare für Vorträge und/oder Beratertätigkeit bzw. Fortbildungsunterstützung erhalten: Boehringer Ingelheim, Mylan, Dexcom, Abbott. L. Stechemesser: Vortragshonorare und Honorare für Beratungstätigkeit von folgenden Firmen: Abbott, Amgen, Astra Zeneca, Boehringer Ingelheim, Dexcom, Eli Lilly, Medtronic, Medtrust, MSD, Mylan, Novartis, NovoNordisk, Roche Diabetes Care, Sanofi Aventis. V. Parzer, M. Wakolbinger und B. Itariu geben an, dass kein Interessenkonflikt besteht.
